# Anemoside B4 attenuates abdominal aortic aneurysm by limiting smooth muscle cell transdifferentiation and its mediated inflammation

**DOI:** 10.3389/fimmu.2024.1412022

**Published:** 2024-05-31

**Authors:** Shuhan Chu, Dan Shan, Luling He, Shilin Yang, Yulin Feng, Yifeng Zhang, Jun Yu

**Affiliations:** ^1^ Center for Translational Medicine, Jiangxi University of Chinese Medicine, Nanchang, Jiangxi, China; ^2^ Department of Cardiovascular Sciences and Center for Metabolic Disease Research, Lewis Katz School of Medicine, Temple University, Philadelphia, PA, United States; ^3^ National Pharmaceutical Engineering Center (NPEC) for Solid Preparation in Chinese Herbal Medicine, Nanchang, Jiangxi, China

**Keywords:** Anemoside B4, abdominal aortic aneurysm, vascular smooth muscle cell, transdifferentiation, vascular inflammation

## Abstract

Abdominal aortic aneurysm (AAA) is a degenerative disease characterized by local abnormal dilation of the aorta accompanied by vascular smooth muscle cell (VSMC) dysfunction and chronic inflammation. VSMC dedifferentiation, transdifferentiation, and increased expression of matrix metalloproteinases (MMPs) are essential causes of AAA formation. Previous studies from us and others have shown that Anemoside B4 (AB4), a saponin from *Pulsatilla chinensis*, has anti-inflammatory, anti-tumor, and regulatory effects on VSMC dedifferentiation. The current study aimed to investigate whether AB4 inhibits AAA development and its underlying mechanisms. By using an Ang II induced AAA model *in vivo* and cholesterol loading mediated VSMC to macrophage transdifferentiation model *in vitro*, our study demonstrated that AB4 could attenuate AAA pathogenesis, prevent VSMC dedifferentiation and transdifferentiation to macrophage-like cells, decrease vascular inflammation, and suppress MMP expression and activity. Furthermore, KLF4 overexpression attenuated the effects of AB4 on VSMC to macrophage-like cell transition and VSMC inflammation *in vitro*. In conclusion, AB4 protects against AAA formation in mice by inhibiting KLF4 mediated VSMC transdifferentiation and inflammation. Our study provides the first proof of concept of using AB4 for AAA management.

## Introduction

Abdominal Aortic Aneurysm (AAA) is a condition in which the wall of the abdominal aorta becomes less elastic, and the degradation of the elastic fibers and collagen leads to dilatation of the abdominal aorta. AAA is diagnosed when the abdominal aorta is dilated by more than 50% in diameter (1). With an 80% mortality rate due to a ruptured aneurysm, this condition poses a significant threat to patients’ health (2). Current treatment relies on surgical intervention for those with a diameter greater than 5.0 cm in women and 5.5 cm in men (3). Therefore, it is significant to uncover the mechanisms of the pathogenesis of AAA and develop therapeutic agents for preventing and treating AAA.

The main pathophysiology of AAA includes chronic inflammation of the aortic wall, vascular smooth muscle cell (VSMC) phenotypic transition, and degradation of extracellular matrix (ECM) (4). VSMCs are the predominant constituents of the arterial walls and are essential in maintaining the vessel structure and remodeling process. They are remarkably plastic cells that display a physiologically quiescent and contractile phenotype but retain the ability to re-enter the cell cycle following injury (5, 6). Numerous studies have demonstrated that phenotypic transformation, including dedifferentiation and transdifferentiation, and apoptosis of VSMCs play essential roles in the pathogenesis of AAA (7–10). One type of VSMC transdifferentiation refers to converting VSMCs into macrophage-like cells with the same functional structure as macrophages under pathological conditions. In recent years, an increasing number of studies have demonstrated that VSMC to macrophage-like cell transdifferentiation contributes to the development of AAA and atherosclerosis (11–14). Therefore, a therapeutic agent attenuating VSMC transdifferentiation could be a practical approach for managing AAA.

A critical question is what regulates the transdifferentiation of VSMCs. Krüppel-like factor 4 (KLF4) is the most well-recognized driver of synthetic VSMC (15) and has been shown to contribute to the development of cardiovascular diseases, including AAA (11, 16–18). Shankman et al. demonstrated that in a model of cholesterol-induced VSMC transdifferentiation, the knockdown of KLF4 significantly reduced the mRNA expression of the macrophage marker Lgals3 and lipid uptake of the cells, suggesting an essential role of KLF4 in VSMC transdifferentiation (19). However, whether VSMC transdifferentiation also regulates MMP activity, another critical factor of AAA development, remains to be elucidated.

*Pulsatilla chinensis* (*P. chinensis*) is the dried root of the buttercup plant Pulsatilla, which is clinically used for anti-inflammation, anti-oxidation, and immune regulation (20, 21). According to the Chinese Pharmacopoeia, saponin Anemoside B4 (AB4) is the main chemical component (more than 4.6%) and has been used as the standard of the quality control of *P. chinensis* (22). Numerous studies have shown that AB4 is the primary pharmacologically active ingredient of *P. chinensis*. Previous studies on AB4 have mainly focused on its anti-inflammation (17, 23, 24) and anti-cancer (25, 26) effects. Notably, we have shown that AB4 inhibits VSMC cell proliferation and migration and attenuates neointimal hyperplasia (27). However, the mechanism by which AB4 modulates VSMC plasticity, particularly transdifferentiation, remains elusive, and its potential efficacy in mitigating the development of AAA remains an open question. Herein, using Ang II induced mouse AAA model *in vivo* and cholesterol induced VSMC transdifferentiation *in vivo*, our results demonstrated that AB4 prevented VSMC dedifferentiation and the transdifferentiation into macrophage-like cells induced by water-soluble cholesterol. AB4 preserved the morphology and function of VSMCs through a KLF4 dependent mechanism and reduced inflammation and MMP activity. Consequently, these actions attenuated AAA progression *in vivo*.

## Materials and methods

### Experimental animals

Eight to 10-week-old male ApoE^-/-^ mice (Hunan Slake Kingda Laboratory Animal Co.) were housed at the Animal Center of Jiangxi University of Traditional Chinese Medicine. These mice were provided ad librium access to chow and water in humidity-controlled rooms under a 12 h light/dark cycle. Experimental protocols were approved by the Institutional Animal Care & Use Committee of the Jiangxi University of Chinese Medicine and the Animal Welfare & Ethics Committee of the Jiangxi University of Traditional Chinese Medicine. All experiments followed the Chinese guidelines for laboratory animal welfare and ethics.

### Animal model

Ang II-induced AAA model was established as previously described (28). Briefly, mice were anesthetized by inhalation of isoflurane, and mini-osmotic pumps (Alzet 2004, Shanghai Yuanmai Biotechnology Co., Ltd.) filled with 1000 ng/kg/min Ang II (model group & AB4 treatment group) or normal saline (control group) were implanted subcutaneously. Mice received daily intraperitoneal injections of normal saline (model group) or AB4 solution (AB4 treatment group, 10 mg/kg/day) for 28 days.

### Doppler ultrasound evaluation of aortic dilation

Small animal ultrasound (Vevo2100, Visual Sonics) was used to monitor aortic expansion in mice 1 day before surgery and at 7-, 14-, 21- and 28- days post-surgery. Isoflurane inhalation was used for anesthesia throughout the ultrasound process. The anesthetized mice lay flat to expose their abdomen. Following skin preparation, a coupling agent was applied. The MS-550 probe was used to determine the largest abdominal aorta of the mouse in a longitudinal section from top to bottom. Then, the probe was rotated 90° for cross-sectional scanning. Longitudinal and transverse ultrasound images were obtained, and the maximum diameters of the aortae were quantified using Vevo LAB (Visual Sonics).

### VSMCs isolation and culture

Primary VSMCs were obtained from the thoracic aortas of 6–8-week-old C57BL/6 mice (Hunan Slake Kinda Laboratory Animal Co.) as previously described (29). The thoracic aortae were harvested from fully anesthetized mice and digested with a digestion solution containing collagenase type II (Worthington; 175 units/ml) and elastase (Sigma; 1.25 units/ml) for 30 min at 37°C. After adventitia was removed, the aortae were cut into 1–2 mm lengths and further digested with a solution containing collagenase type II (175 units/ml) and elastase (2.5 units/ml) for 60 min at 37 °C. The cell suspension was centrifuged at 1000 rpm for 3 min. Cells were incubated in DMEM containing 20% FBS, 100 μg/ml penicillin, and 100 μg/ml streptomycin at 37°C and 5% CO_2_. Cells from generations 4–7 were used for all experiments. The VSMCs were starved with DMEM-F12 medium (0.2% FBS) for 24 hrs before experimental treatments. VSMCs were stimulated with 40 μg/ml water-soluble cholesterol (Sigma, C4951) for 72 hrs to induce transdifferentiation (30).

### Recombinant adenovirus construction and expression

The adenovirus that expresses KLF4 (Ad-KLF4) was constructed and purified using the Admax system (Hanbio Biotechnology, Shanghai, China). Ad-KLF4 infection at 100 MOI for up to 72 hrs achieved VSMC overexpression of KLF4.

### Total RNA extraction and quantitative real-time PCR

Total RNA was isolated using Trizol (Takara) according to the manufacturer’s protocol. The concentration of RNA was determined using a nanodrop spectrophotometer (Thermo Fisher Scientific, USA). One μg of total RNA from each sample was reverse transcribed into cDNA using a reverse transcription kit (Yisheng Biology, China). The transcribed cDNA was then subjected to a quantitative real-time polymerase chain reaction (qRT-PCR) using the SYBR Green PCR kit (Yisheng Biotech China). The mRNA levels were normalized to those of 18s mRNA. Primers specific to each of the genes tested were synthesized by Biotech. The primer sequences are shown in **Supplementary Table 1**.

### Western blot

Mouse aortae or VSMCs were lysed with RIPA buffer, and the tissue was disrupted with a homogenizer (aorta) or a sonicator (VSMC) and then lysed on ice for 15 mins. Tissue or cell lysate was centrifuged at 12,000 × g for 20 mins at 4°C. The supernatant was quantified using a BCA kit (Kang Wei Century, China). Equal amounts of total protein were loaded onto SDS-PAGE gels and transferred onto nitrocellulose membranes. After transfer, the membrane was blocked with 5% BSA in TBST solution for one hour at room temperature. And then incubated with primary antibodies overnight at 4°C. The antibodies used are listed in **Supplementary Table 2**. Membranes were then probed with species-appropriate secondary antibodies for 1 hr at room temperature. The specific immunoreactive protein bands were detected by an Enhanced Chemiluminescence (ECL) kit (Kang Wei Century, China) or a Gel DocTM XR+ System using Image Lab software (BioRad) and analyzed using Image J (National Institutes of Health, USA).

### Histology and immunofluorescence staining

The entire aorta was excised under a stereomicroscope after the mice were euthanized and perfused with PBS solution. Sections of the suprarenal abdominal aorta were cut into 8 μm-thick frozen sections. Sections of the abdominal aorta were subjected to Hemotoxiline and Eosin (H&E, Nanchang Yulu, China), Verhoeff-Van Gieson (VVG, DC0045, Leagene, China), and Masson-Trichrome staining (DC0033, Leagene, China) for histoarchitectural evaluation of the aneurysms. For immunofluorescent staining, frozen sections of suprarenal abdominal aortic sections were incubated with antibodies to specifically against MMP2, MMP9, α-SMA, and CD68 (**Supplementary Table 2**), followed by the secondary Alexa Fluor 488-conjugated donkey anti-rat IgG (1:400, A-21208, Invitrogen, Eugene, OR, USA), Alexa Fluor 594 goat anti-rabbit IgG (1:500, R37117, Invitrogen USA), Alexa Fluor 594 goat anti-Rat IgG (1:500, A11007, Invitrogen USA), and the nuclei were stained with DAPI (BD5010, Bioworld, USA).

### MMP activity assay - gelatin zymography

Gelatin zymography was performed as described (31). Briefly, equal amounts of aortic protein samples were separated on 10% SDS-PAGE gels with 0.1% gelatin as substrate. Then, wash the gel with buffer containing 2.5% Triton X-100, 50 mM Tri-Hcl (PH=7.5), 5 mM CaCl_2_, 1 μM ZnCl_2_ for 5–10 min. After incubation for 24 hrs at 37°C in incubation buffer containing 50 mM Tris-HCl (PH=7.8), 1 μM ZnCl_2_, 5 mM CaCl_2_, 1% Triton X-100 and 0.02% Brij 35 (Merk). Staining was then performed with 0.05% Coomassie Brilliant R-250 dye (Merk, 1125530025, USA) for 30 min. Decolorize with the destaining solution (40% methanol, 10% acetic acid) until bright white bands appear. To examine MMP activity in animal samples, unfixed aortic sections were air-dried at room temperature for 30 min, incubated with DQ™ porcine skin gelatin-fluorescein conjugate (D12054, Thermo Fisher, USA) at 37°C for 1.5 hrs, and washed with PBS 5 mins for three times. The nuclei were stained with DAPI. The green fluorescence was then detected using fluorescence microscopy.

### Statistical analysis

Values are presented as mean ± SEM from at least three independent Experiments. GraphPad Prism Software version 8.0 was used for statistical analysis. One-way ANOVA followed by the Tukey post-test to correct for multiple comparisons was used to perform multiple group comparisons. A two-tailed Student’s-test was used to analyze the differences between the two groups. P < 0.05 were considered statistically significant.

## Result

### AB4 protects against Ang II induced AAA formation *in vivo*

**Figure 1:**
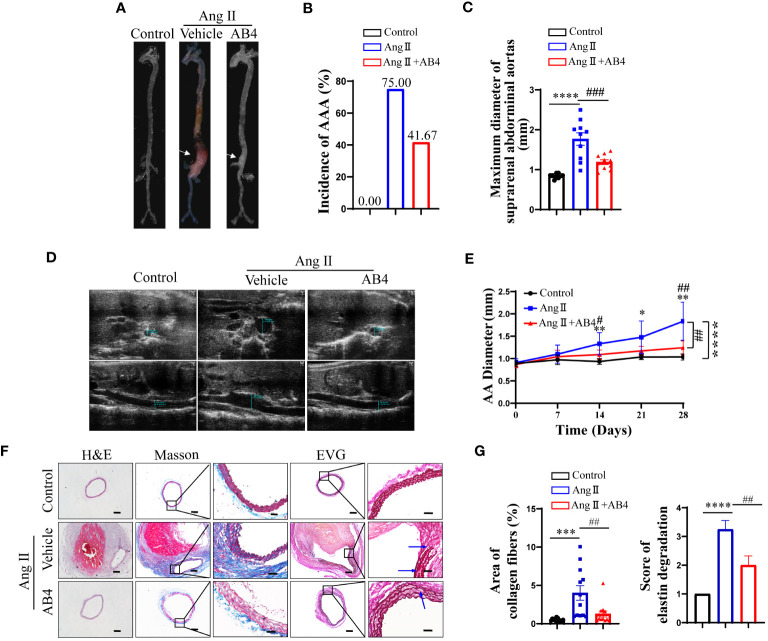
AB4 inhibits the pathogenesis of Ang II-induced AAA mice. **(A)** Representative images of AAA; **(B)** AAA incidence; **(C)** Maximal diameter of the AAA; **(D)** Representative images of Doppler ultrasound in each group after 28 days; **(E)** The growth curve of the AAA among the three groups; **(F, G)** Representative H&E, EVG, and Masson trichrome staining images of abdominal aorta in three groups (The blue arrow indicates elastic fiber breakage, n=12). The scale bar in columns 1, 2, and 4 indicates 200 μm; The scale bar in columns 3 and 5 indicates 50 μm. Statistical differences were assessed by one-way ANOVA analysis of variance test. Data shown are means ± SEM. *P < 0.05, **P < 0.01, ***P < 0.001, ****P < 0.0001 vs. Control group; ^##^P < 0.01, ^###^P < 0.001 vs. Ang II (Vehicle) group.

To evaluate the effect of AB4 on experimental AAA, Ang II was delivered at a dose of 1000 ng/kg/min via a subcutaneously implanted micro-osmotic pump to ApoE^-/-^ mice. After 28 days of Ang II infusion, AAA occurred in the Ang II group with an incidence of 75.00% (9/12), and AB4 treatment inhibited the development of AAA with a decreased incidence of 41.67% (5/12) (**Figures 1A, B**). This result was confirmed by the measurement of the maximal outer diameter of AA *ex vivo*, showing that the AB4-treated group significantly reduced suprarenal AA diameter compared to the vehicle-treated model group (**Figure 1C**). Serial doppler ultrasound imaging analysis showed that the suprarenal AA expansion was significantly inhibited in the AB4 treatment group compared with the model group (**Figures 1D, E** **).** Histologically, we performed H&E, Masson-Trichrome, and Elastin and Van Gieson (EVG) staining on the crosssections of AAA samples and observed that intraluminal thrombus was formed, aortic adventitial thickened and matrix remodeled, collagen fibers were deposited, and elastic fibers were broken and degenerated, after 4 weeks of Ang II infusion. However, these pathological features were alleviated after administration of AB4 (**Figures 1F, G**). These results indicated that AB4 attenuated the pathogenesis of AAA *in vivo*.

### AB4 maintains VSMC contractile gene expression and decreases CD68^+^ macrophage numbers in the aortae *in vivo*

**Figure 2:**
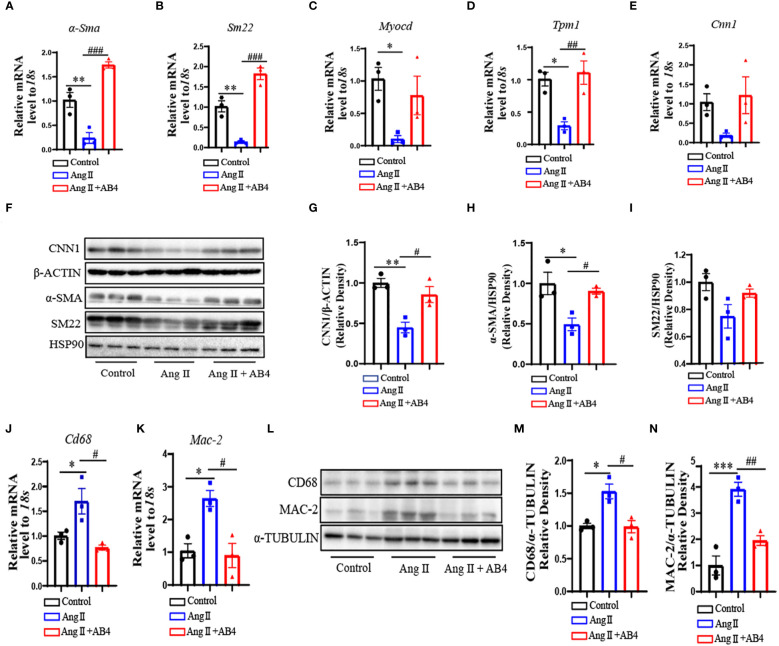
AB4 inhibits VSMC to macrophage-like cell transdifferentiation *in vivo*. **(A–E)** The relative mRNA levels of *α-Sma*, *Sm22*, *Myocd*, *Tpm1* and *Cnn1* in the aortae were measured by qRT-PCR (n=3); **(F)** CNN1, α-SMA, and SM22 protein levels were measured using Western blot; **(G–I)** CNN1, α-SMA and SM22 protein levels were quantified by densitometry analysis (n=3); **(J, K)** The relative mRNA levels of *Cd68* and *Mac-2* in the aortae were measured by qRT-PCR (n=3); **(L)** CD68 and MAC-2 protein levels were measured using Western blot; **(M, N)** CD68 and MAC-2 protein levels were quantified by densitometry analysis (n=3); Statistical differences were assessed by one-way ANOVA analysis of variance test. Results are presented as the mean ± SEM. *P < 0.05, **P < 0.01, ***P < 0.001 vs. Control group; ^#^P < 0.05, ^##^P < 0.01, ^###^P < 0.001 vs. Ang II (Vehicle) group.

VSMC to macrophage-like cell transdifferentiation promotes AAA and atherosclerosis pathogenesis (32–34). Our previous study has shown AB4 attenuated VSMC dedifferentiation (27). To investigate whether AB4 inhibits VSMC transdifferentiation in Ang II induced AAA *in vivo*, we examined the expression of VSMC contractile genes in aortae. Ang II stimulation significantly down-regulated the mRNA expression of alpha-smooth muscle actin (*α-Sma*), SM22 alpha (*Sm22*), myocardin (*Myocd*), Tropomyosin 1 (*Tpm1*), and Calponin (*Cnn1*) (**Figures 2A–E**). AB4 treatment significantly reversed these effects. Western Blot analysis showed that the contractile protein level decreased in the aortae after Ang II stimulation, and the AB4 treatment reversed these effects *in vivo* (**Figures 2F–I**). Meanwhile, Ang II induced aortic macrophage markers CD68 and Galectin-3 (MAC-2) gene and protein expression, an effect significantly attenuated by AB4 treatment (**Figures 2J–N**). To determine whether the increased expression of macrophage marker genes is attributed to the transdifferentiation of VSMCs. The co-localization of CD68 and α-SMA was examined by immunofluorescence staining on frozen sections of the aortae. Compared with the control group, the expression of α-SMA in the aortic wall of the Ang II group was significantly reduced, and a large amount of co-localization of α-SMA and CD68 was observed, suggesting a potential VSMC to macrophage-like cell transdifferentiation. Notably, AB4 effectively reduced α-SMA/CD68 double positive cell numbers (**Supplementary Figure 1**). Taken together, these results suggest that AB4 preserved VSMCs contractile phenotype and possibly reduced VSMC to macrophage-like cell transdifferentiation in the vascular wall in the Ang II induced AAA *in vivo*.

### AB4 reduces MMP2 and MMP9 expression in the aneurysm wall

**Figure 3:**
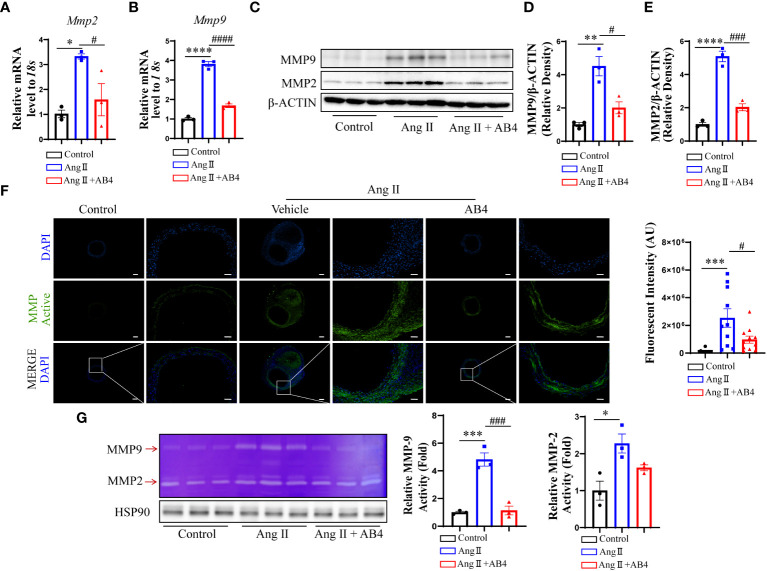
Effect of AB4 treatment on aortic MMP2 and MMP9 *in vivo*. **(A, B)** The relative mRNA levels of *Mmp2* and *Mmp9* in the aortae were measured by qRT-PCR (n=3); **(C)** MMP9 and MMP2 protein levels were examined by Western blot; **(D, E)** MMP-9 and MMP-2 protein levels were quantified by densitometry analysis (n=3); **(F)** Representative *in situ* gel zymograms for cryostat section (n=12); **(G)** Representative gelatin zymography of abdominal aortas protein from the indicated groups and quantified by densitometry analysis (n=3); The scale bar in columns 1, 3 and 5 indicates 200 μm; The scale bar in columns 2, 4 and 6 indicates 50 μm. Statistical differences were assessed by one-way ANOVA analysis of variance test. Results are presented as the mean ± SEM. *P < 0.05; **P < 0.01; ***P <0.001; ****P < 0.0001 vs. Control group; ^#^P < 0.05; ^###^P < 0.001; ^####^P < 0.0001 vs. Ang II (Vehicle) group.

It has been reported that dedifferentiated and transdifferentiated VSMCs produce more MMPs, especially MMP2 and MMP9, which play a vital role in AAA development (35, 36). We examined the mRNA levels of *Mmp2* and *Mmp9* in the aneurysm tissue. As shown in **Figures 3A, B**, MMP2 and MMP9 were significantly elevated in the model group compared to the control, an effect significantly reversed by AB4 treatment. Similar results were also observed at the protein level by western blotting (**Figures 3C–E**) and by immunofluorescence staining (**Supplementary Figure 2**). To further determine the effect of AB4 on MMP activity *in vivo*. The enzyme activities of MMP2 and MMP9 were detected by gelatin zymography. *In situ* zymography on AAA sections (**Figure 3F**) and gelatin zymography on aortic wall lysate (**Figure 3G**) showed that AB4 effectively attenuated Ang II induced MMP activity *in vivo*. These results indicate that MMP2 and MMP9 expression and activity are elevated in AAA development, and AB4 may prevent AAA formation by inhibiting MMP activity.

### Animoside B4 inhibits vascular inflammation

**Figure 4:**
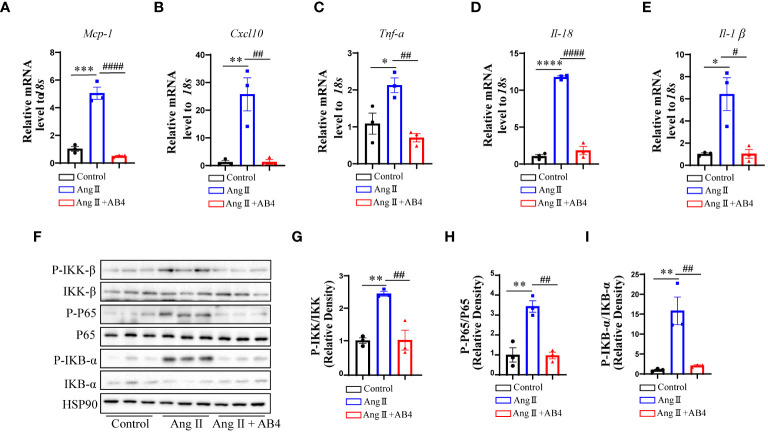
Effects of AB4 on the NF-κB signaling *in vivo*. **(A–E)** The relative mRNA levels of inflammatory cytokines *Mcp-1*, *Cxcl10*, *Tnf-α*, *Il-18* and *Il-1β* in the aortae were measured by qRT-PCR (n=3); **(F)** NF-κB signaling pathway protein levels were examined by western blotting; **(G–I)** NF-κB pathway protein levels were quantified by densitometry analysis (n=3); Statistical differences were assessed by one-way ANOVA analysis of variance test. Results are presented as the mean ± SEM. *P < 0.05; **P < 0.01; ****P < 0.0001 vs. Control group; ^#^P < 0.05; ^###^P < 0.001 vs. Ang II (Vehicle) group.

VSMC plasticity and the ability to perform nonprofessional phagocytic functions are critical phenomena maintaining the inflammatory state. Chronic and excessive activation of vascular inflammatory factors is a crucial cause in the pathogenesis of AAA (37–39). We thus asked whether AB4 could attenuate the activation of vascular inflammation, thereby inhibiting the progression of AAA. Our qRT-PCR results showed that after Ang II infusion for 4 weeks, the expression of inflammatory genes (*Mcp-1, Cxcl10, Tnf-α, Il-18*, and *Il-1β)* significantly increased in the aorta compared with the control group. After AB4 treatment, the expression of vascular inflammation genes was reduced (**Figures 4A–E**). NF-κB is one of the major transcription factors regulating above mentioned pro-inflammatory factors (40). Therefore, we next investigated the effect of AB4 on NF-κB signaling pathway. Ang II induced elevation of IKK-β, IKB-α, and NF-κB phosphorylation were significantly suppressed by AB4 treatment (**Figures 4F–I**) in aortae. To examine whether these effects are due to decreased VSMC inflammation, we stimulated VSMCs with cholesterol *in vitro*. As shown in **Supplementary Figure 3**, cholesterol activated IKK-β, IKB-α, and NF-κB phosphorylation, the effects were suppressed by AB4 treatment. Similarly, AB4 treatment decreased the inflammatory cytokine *Mcp-1* and *Il-6* mRNA expression induced by cholesterol stimulation. Taken together, our results suggest that the inhibitory effect of AB4 on AAA progression may be related to the regulation of the IKK/IKB/NF-κB signaling pathway in VSMC.

### AB4 inhibits VSMC to macrophage-like cell transdifferentiation *in vitro*

**Figure 5:**
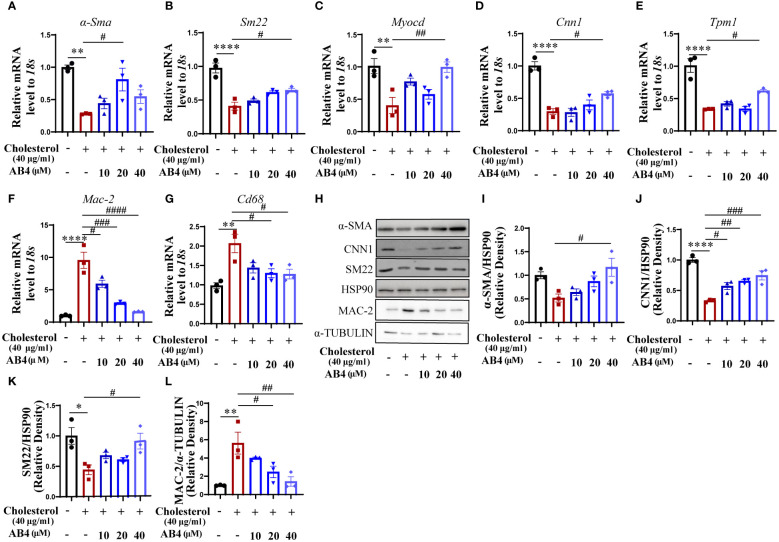
AB4 inhibits VSMC to macrophage-like cell transdifferentiation *in vitro*. **(A–G)** qRT-PCR determination of smooth muscle marker genes **(H–K)** and macrophage marker gene **(L)** expression in SMCs (n=3); **(H)** The expression levels of contractile protein SM22-α, α-SMA, CNN1 and MAC-2 were measured using Western blot; **(I–L)** SM22, α-SMA, CNN1 and MAC-2 protein levels were quantified by densitometry analysis (n=3); Statistical differences were assessed by one-way ANOVA analysis of variance test. *P < 0.05; **P < 0.01; ***P < 0.001; ****P < 0.0001 vs. Control group. ^#^P < 0.05; ^##^P < 0.01; ^###^P < 0.001; ^####^P < 0.0001 vs. Cholesterol group.

To directly examine whether AB4 inhibits VSMC transdifferentiation, we used a well-established cholesterol stimulation model (30). VSMCs were stimulated with 40 µg/ml of water-soluble cholesterol for 72 h to induce transdifferentiation. As shown in **Figures 5A–G**, cholesterol promotes VSMC transdifferentiation, as evidenced by reduced mRNA expression of contractile genes (*α-Sma, Sm22, Myocd, Cnn1*, and *Tpm1*) and increased macrophage markers *Cd68* and *Mac-2*. Also, the protein levels of α-SMA, CNN1, SM22 were increased, while MAC-2 was decreased (**Figures 5H–L**) in response to cholesterol loading. Notably, AB4 reversed the above phenomenon. Transdifferentiated VSMCs have lipid uptake potential (41, 42). We further determined the effect of AB4 on VSMC lipid uptake. Oil red O staining showed that AB4 reduced lipid accumulation in VSMC dose dependently (**Supplementary Figures 4A, B**). To more intuitively observe the transdifferentiation of VSMCs into macrophage-like cells, we also carried out co-localization immunofluorescence staining of α-SMA and CD68 on VSMCs. As shown in **Supplementary Figure 4C**, the fluorescence intensity of α-SMA decreased, and CD68 increased after cholesterol stimulation. AB4 treatment dose-dependently preserved α-SMA expression and prevented CD68 upregulation in VSMCs. Taken together, these results suggest AB4 effectively inhibits cholesterol-induced VSMC transdifferentiation into macrophage-like cells.

### AB4 reduces cholesterol-induced expression of MMP2 and MMP9 in VSMCs

**Figure 6:**
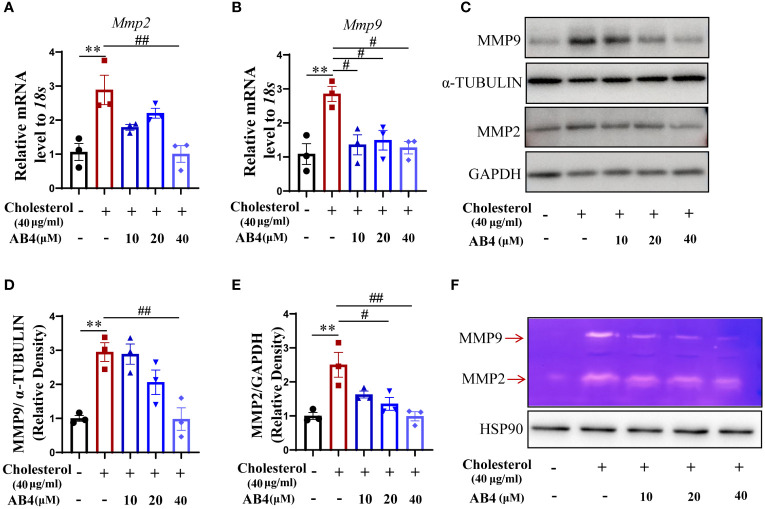
AB4 decreases cholesterol-induced VSMC MMP2 and MMP9 expression and activity. **(A, B)** VSMCs were pretreated with AB4 for 24 h, followed by water-soluble cholesterol (40 μg/ml) stimulation for 48 h. The relative mRNA levels of *Mmp2* and *Mmp9* in VSMCs were measured by qRT-PCR (n=3); **(C)** MMP9 and MMP2 protein levels in VSMCs were examined by Western blot; **(D, E)** MMP9 and MMP2 protein levels were quantified by densitometry analysis (n=3); **(F)** The protein activities of MMP-9 and MMP-2 in different groups were determined by gelatin zymography; Statistical differences were assessed by one-way ANOVA analysis of variance test. Results are presented as the mean ± SEM. **P < 0.01 vs. Control group; ^#^P < 0.05; ^##^P < 0.01 vs. Cholesterol group.

We demonstrated that AB4 inhibits the aortic MMP2 and MMP9 expression in mouse AAA samples *in vivo* (**Figure 3**). However, the cellular component that contributed to this was unclear. To address this, we stimulated VSMCs with water-soluble cholesterol to undergo transdifferentiation, and MMP expression and activity were examined. The results show that the mRNA and protein expression levels of MMP2 and MMP9 were elevated in response to cholesterol stimulation, and AB4 could inhibit *Mmp2* and *Mmp9* mRNA (**Figures 6A, B**) and protein expression (**Figures 6C–E**). Meanwhile, gelatin zymography showed that AB4 reversed the cholesterol-induced increase of MMP activity in cholesterol-stimulated VSMCs (**Figure 6F**). These results suggested that during VSMC transdifferentiation, MMP2 and MMP9 were upregulated and activated, a phenomenon has not been reported before. AB4 inhibited the transdifferentiation of VSMCs while suppressing MMP2 and MMP9 expression and activity.

### AB4 inhibits KLF4 mediated VSMC to macrophage-like cell transdifferentiation

**Figure 7:**
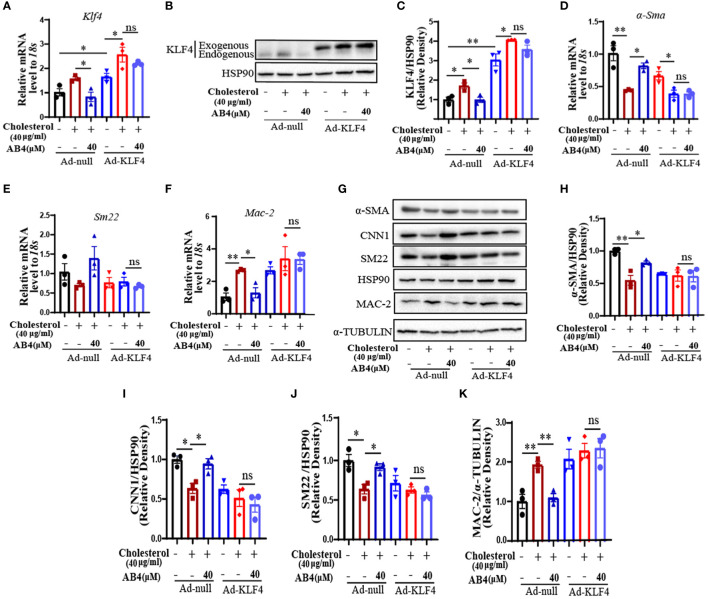
AB4 attenuates VSMC transdifferentiation by regulating KLF4. **(A)** The relative mRNA level of *Klf4* after ad-null or ad-KLF4 infection in VSMCs was determined by qRT-PCR (n=3); **(B)** After overexpression of KLF4 by infection with Ad-KLF4, VSMCs were exposed to cholesterol in presence or absence of AB4, and KLF4 protein level in VSMCs was determined by Western blot; **(C)** KLF4 protein level were quantified by densitometry analysis (n=3); **(D–G)** The relative mRNA levels of *α-Sma*, *Sm22* and *Mac-2* in VSMCs were examined by qRT-PCR after overexpression of KLF4 (n=3); **(H)** α-SMA, CNN1, SM22 and MAC-2 protein levels in VSMCs were examined by Western blot analysis after KLF4 overexpression; **(I–K)** α-SMA, CNN1, SM22 and MAC-2 protein levels were quantified by densitometry analysis (n=3); Statistical differences were assessed by one-way ANOVA analysis of variance test. Results are presented as the mean ± SEM. *P < 0.05; **P < 0.01.

KLF4 plays an important role in VSMC plasticity, and can regulate VSMC phenotypic transformation, foaming, transdifferentiation, proliferation, migration, etc., in disease states, and then accelerate the development of the disease (19, 28, 43, 44). It has been shown that KLF4 deficiency inhibited AAA formation in mice (18). To investigate whether KLF4 is involved in AB4 attenuated VSMC transdifferentiation and AAA development, we first examined the KLF4 expression in AAA samples *in vivo*. As shown in **Supplementary Figure 5A**, AB4 significantly inhibited the increase of *Klf4* mRNA in the aortae. At the protein level, KLF4 was dramatically elevated in early (1 week) AAA development and lasted till 4 weeks after Ang II stimulation (**Supplementary Figures 5B–D**). AB4 treatment significantly inhibited the KLF4 upregulation at both mRNA (**Supplementary Figure 5A**) and protein (**Supplementary Figures 5B–D**) levels. Similarly, KLF4 mRNA and protein levels were increased during cholesterol-induced VSMCs transdifferentiation into macrophage-like cells, and AB4 attenuated KLF4 upregulation dose-dependently (**Supplementary Figures 5E–G** **).** To determine whether AB4 attenuates VSMC transdifferentiation by regulating KLF4, we performed “rescue” experiments by overexpressing KLF4. VSMCs were infected with Ad-null or Ad-KLF4 and treated with or without AB4 in the presence or absence of cholesterol stimulation. **Figures 7A–C** showed that KLF4 expression was markedly increased after Ad-KLF4 infection, suggesting a successful overexpression. Importantly, KLF4 overexpression reversed the protective effect of AB4 on cholesterol induced VSMC to macrophage-like cell transdifferentiation, as evidenced by VSMC contractile gene (*α-Sma and Sm22)* and macrophage gene (*Mac-2*) mRNA (**Figures 7D–F**) and protein expression (**Figures 7G–K**). These results indicated that AB4 regulated VSMC transdifferentiation by regulating KLF4. However, AB4 treatment still decreased *Mmp2* and *Mmp9* transcription under KLF4 overexpression conditions (**Supplementary Figure 6**), suggesting AB4 suppresses cholesterol induced MMP transcription, at least in part, in a KLF4 independent manner in VSMC.

## Discussion

**Figure 8:**
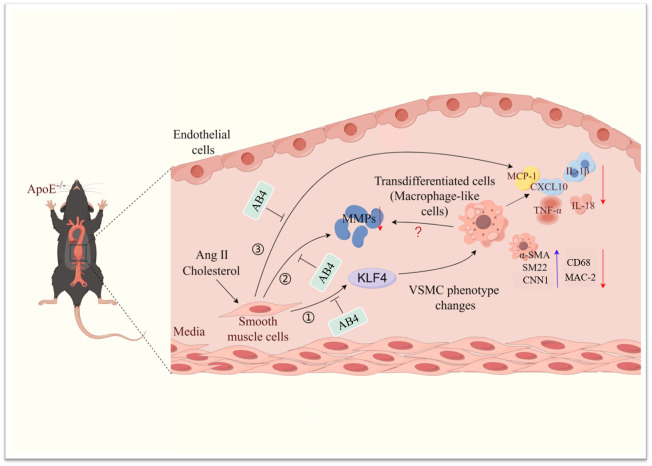
Schematic illustration of the effect of AB4 on VSMC plasticity and AAA formation. AB4 can prevent the occurrence and development of Ang II induced AAA in ApoE^-/-^mice by maintaining smooth muscle cell homeostasis, reducing the overexpression of MMP activity and content, and inhibiting the expression of inflammatory factors. At the mechanistic level, AB4 can inhibit VSMC transdifferentiation by inhibiting the overexpression of KLF4 in VSMC. It can also reduce the expression of MMPs and inflammatory factors.

AAA is a common aortic disease with high mortality (45), which is caused by VSMC dedifferentiation, transdifferentiation, apoptosis, increased vascular inflammation, and extracellular matrix degradation (46). Targeting these causal mechanisms thus holds great value in managing AAA. The current study shows that AB4, a saponin from *Pulsatilla chinensis*, (1) attenuated AAA pathogenesis, (2) prevented VSMC dedifferentiation and transdifferentiation to macrophage-like cells, (3) decreased vascular inflammation, (4) decreased MMP expression and activity, and (5) mechanistically, KLF4 overexpression attenuated the effects of AB4 on VSMC to macrophage-like cell transition and VSMC inflammation. (**Figure 8**).

It is well known that VSMC dysfunction is a pathological feature of AAA formation (47). The previous study in our laboratory showed that AB4 could inhibit the proliferation, migration, and dedifferentiation of VSMCs (27). Therefore, we hypothesized that AB4 could achieve AAA prevention by maintaining aortic VSMC homeostasis. To experimentally test this, we used Ang II induced mouse AAA model. AB4 (10 mg/kg/day) was intraperitoneally injected daily after Ang II mini pump implantation. Our results showed AB4 inhibited aortic dilatation and reduced the incidence of AAA (**Figures 1A–E**). After AB4 treatment, intraluminal thrombosis, elastic fiber rupture or degeneration, aortic adventitia thickening, and collagen fiber deposition in the abdominal aorta significantly improved (**Figures 1F, G**). These results suggest that AB4 has a pronounced preventive effect on AAA development, which provides a basis for its clinical translation potential in managing AAA.

VSMCs are the main component of the vascular media, and healthy VSMCs remain static and contract to maintain the elasticity and tension of blood vessels (48). When subjected to pathological stimuli, VSMCs undergo migration, transformation, and proliferation. Among the transformations are synthetic, foamy, osteoblastic, and macrophage-like cells (49, 50). Maintaining the morphology and function of VSMC is a key factor in treating aortic diseases. Sufficient studies have proved that modulating VSMC plasticity, particularly transdifferentiation, is one of the most critical measures for treating aortic diseases such as atherosclerosis and AAA (51–54). Our current study shows that AB4 reduces the Ang II-induced increase in macrophage-like cell numbers in the aorta of ApoE^-/-^ mice and partially restores the expression of VSMC markers (**Figure 2**, **Supplementary Figure 1**). These results indicated that AB4 may attenuate AAA by inhibiting VSMC to macrophage-like cell transdifferentiation. Our *in vitro* study further supports this motion. We chose water-soluble cholesterol (40 μg/ml) to stimulate primary mouse VSMCs for 72 hours [30] as the VSMC transdifferentiation model. The results showed that AB4 treatment significantly attenuated VSMC to macrophage-like cell transdifferentiation and lipid uptake potential (**Figure 5**, **Supplementary Figures 4A, B**). Therefore, we speculate that AB4 protects AAA formation by inhibiting VSMC transdifferentiation *in vivo*. Future studies using the VSMC lineage tracing mouse model are warranted.

Transdifferentiated VSMCs have similar functions as macrophages in pathological states, recruiting inflammatory cells to the aortic wall by secreting various pro-inflammatory cytokines, further exacerbating local inflammation in the aortic wall, and promoting the progression of AAA (46, 55, 56). By examining the expression of inflammatory genes in the aorta, we found that B4 could significantly reduce the expression of pro-inflammatory factors in the AAA (**Figures 4A–E**) and suppress NF-κB signaling pathway activation *in vivo* (**Figures 4F–I**). Based on these results, we speculate that AB4 inhibits VSMC to macrophage-like cell transdifferentiation and thus decreases the inflammation in the vessel wall. The anti-inflammatory effect of AB4 has been documented. It has been reported that AB4 can treat chronic recurrent colitis, acute lung injury, and encephalomyelitis by inhibiting macrophage mediated inflammation (57–59). We thus cannot completely rule out the possibility that AB4 attenuates vascular inflammation by inhibiting innate immune response during AAA formation.

In healthy blood vessels, VSMCs can coordinate the expression of MMPs/TIMPs to maintain the integrity of the ECM and prevent the degradation of elastin and collagen. They also protect the elasticity of the aortic wall, thereby slowing the formation of AAA. MMPs promote ECM degradation during the development of AAA, and the increase in expression is also an essential symbol of AAA development (54, 60–62). MMP2 and MMP9 are the key MMPs that contribute to the formation and development of AAA (63). Here, we demonstrated that AB4 not only prevented the increase in MMP2 and MMP9 expression induced by Ang II but also significantly attenuated the MMP activity (**Figure 3**). In AAA, VSMCs undergo a phenotypic transformation, and the production of MMPs is also enhanced (64, 65). However, whether the production of MMPs is attributed to the transdifferentiation of VSMC into macrophage-like cells has not been reported. Although the function of the macrophage-like cell phenotype of VSMCs *in vivo* cannot be clearly defined, our *in vitro* study demonstrated, for the first time, that the function of VSMCs transformed into macrophage-like cells after cholesterol loading was manifested in the loss of the shrinkage phenotype of VSMCs, the increase of MMPs content and activity (**Figure 6**).

KLF4 is a transcription factor that regulates VSMC phenotype and function (66). KLF4 negatively regulates the expression of many VSMC differentiation genes or competes with SRF for CArG element binding (67). Meanwhile, KLF4 has been shown to play an essential role in AAA, and specific deletion of KLF4 in VSMCs attenuates AAA formation (18). Our results showed that AB4 inhibited Ang II induced upregulation of KLF4 in aortae (**Supplementary Figure 5**). *In vitro*, we found that when cholesterol induces transdifferentiation of VSMC into macrophage-like cells, KLF4 expression levels also increased, and AB4 can reverse this phenomenon. Interestingly, when KLF4 was overexpressed, cholesterol stimulation did not further aggravate the transdifferentiation of VSMC. The VSMC contractile and macrophage marker levels were similar to cholesterol-stimulated (Ad)-null-infected VSMCs, suggesting KLF4 is the major transcription factor regulating VSMC transdifferentiation. These results are consistent with previous reports (19). Importantly, when KLF4 was overexpressed, the protection effects of AB4 on VSMC transdifferentiation were lost (**Figure 7**). Our results strongly indicate that KLF4 is at least one of the major targets of AB4 to inhibit VSMC transdifferentiation. Although AB4 also decreased KLF4 *in vivo*, whether AB4 attenuates AAA through the KLF4 mediated VSMC transdifferentiation *in vivo* needs further investigation.

In conclusion, the current study demonstrates for the first time that AB4 can prevent the development of AAA by regulating the plasticity of VSMCs. AB4 can transcriptionally activate KLF4, participate in the transdifferentiation of VSMC to macrophage transition, and downregulate the expression and activity of MMPs (**Figure 8**). The results of this study provide the basis for the discovery of new drugs and AB4 for the prevention and treatment of AAA in the future.

## Data availability statement

The original contributions presented in the study are included in the article/**Supplementary Material**. Further inquiries can be directed to the corresponding authors.

## Ethics statement

The animal study was approved by Institutional Animal Care & Use Committee of the Jiangxi University of Chinese Medicine and the Animal Welfare & Ethics Committee of the Jiangxi University of Traditional Chinese Medicine. The study was conducted in accordance with the local legislation and institutional requirements.

## Author contributions

SC: Writing – original draft, Visualization, Validation, Methodology, Investigation, Formal analysis, Data curation. DS: Writing – review & editing, Methodology, Data curation. LH: Writing – review & editing, Methodology. SY: Writing – review & editing, Formal analysis. YF: Writing – review & editing, Validation. YZ: Writing – original draft, Validation, Funding acquisition, Conceptualization. JY: Writing – review & editing, Writing – original draft, Supervision, Conceptualization.
