# Supplementary material for: Anemoside B4 attenuates abdominal aortic aneurysm by limiting smooth muscle cell transdifferentiation and its mediated inflammation
Source: Front Immunol. 2024 May 31;15:1412022. doi: 10.3389/fimmu.2024.1412022 (PMC11176519; doi:10.3389/fimmu.2024.1412022)
Supplement: Supplementary file 1 [file DataSheet_1.docx]

**Anemoside B4 attenuates abdominal aortic aneurysm by limiting smooth muscle cell transdifferentiation and its mediated inflammation**

**Shuhan Chu^1,2^, Dan Shan^2^, Luling He**^1^**, Shilin Yang**^3^**, Yulin Feng**^3^**, Yifeng Zhang^1,*^, Jun Yu^2,*^**

^1^Center for Translational Medicine, Jiangxi University of Chinese Medicine, Nanchang 330004, Jiangxi, China

^2^Department of Cardiovascular Sciences and Center for Metabolic Disease Research, Lewis Katz School of Medicine, Temple University, Philadelphia, PA 19140, USA

^3^National Pharmaceutical Engineering Center (NPEC) for Solid Preparation in Chinese Herbal Medicine, Nanchang 330006, Jiangxi, China

***Correspondence:**

Yifeng Zhang, PhD

Center for Translational Medicine,

Jiangxi University of Chinese Medicine, Nanchang 330004, China

Email: [zyf489662913@163.com](mailto:zyf489662913@163.com)

Jun Yu, MD

Department of Cardiovascular Sciences, Center for Metabolic Disease Research, Lewis Katz School of Medicine, Temple University, Philadelphia, PA 19140, USA.

Email: [jun.yu@temple.edu](mailto:jun.yu@temple.edu)

**Supplementary Figure 1**


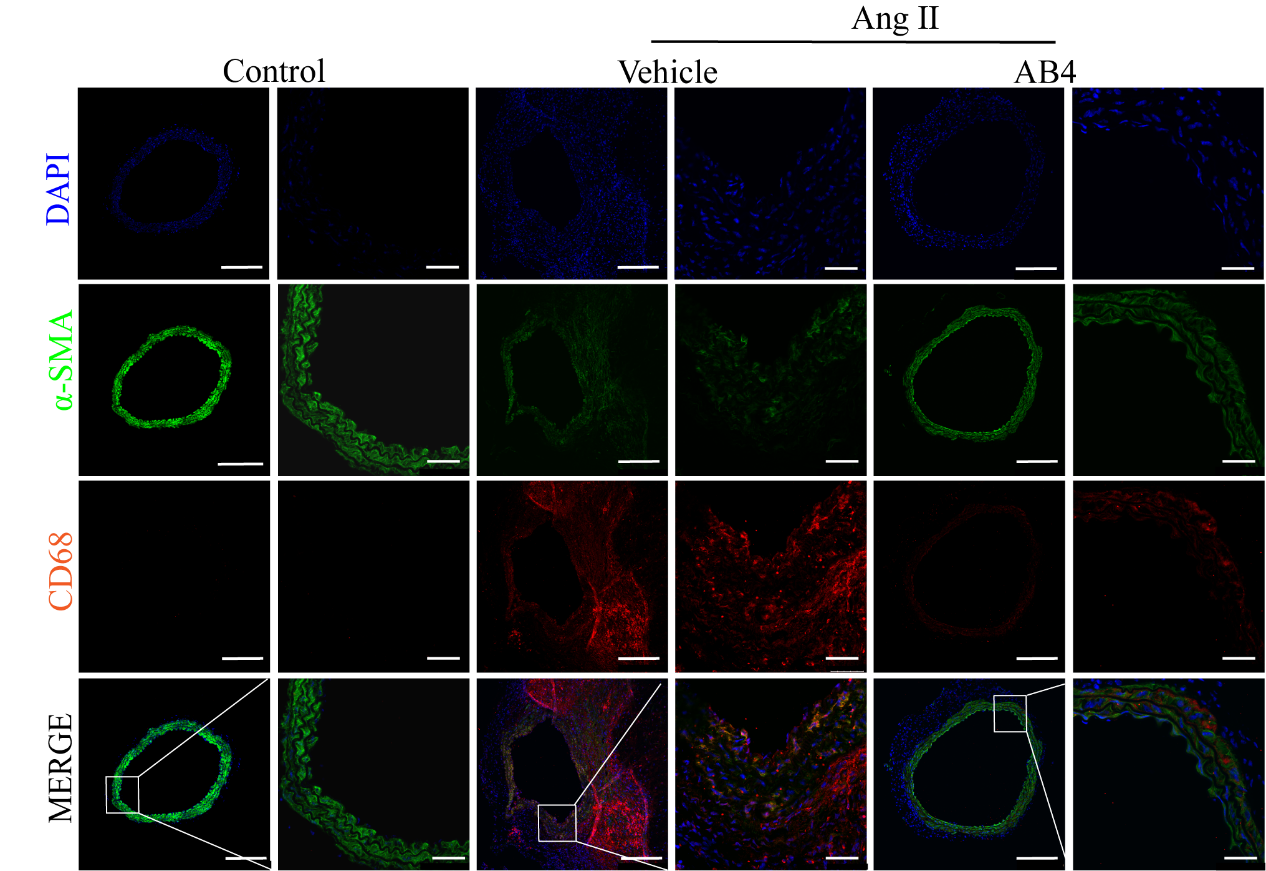


**Supplementary figure 1. AB4 inhibits VSMC to macrophage-like cell transdifferentiation *in vivo*.**

Representative confocal images of immunofluorescence staining of DAPI (blue, top row), VSMC (α-SMA, green, 2^nd^ row), and macrophage (CD68, red, 3^rd^ row) in cross-sections of the suprarenal aorta from each group (n=12). The scale bars in columns 1, 3, and 5 indicate 200 μm, and the ones in columns 2, 4, and 6 indicate 50 μm.

**Supplementary Figure 2**


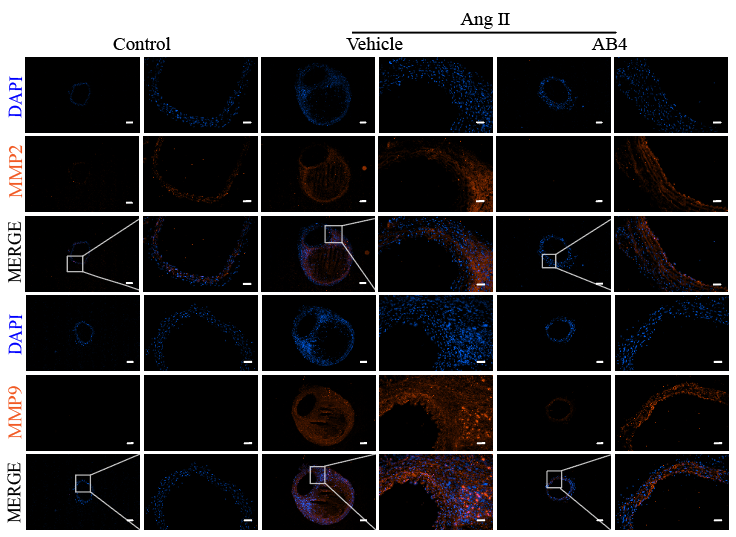


**Supplementary figure 2. Effect of AB4 treatment on matrix metalloproteinases (MMP2 and MMP9) in vivo.**

Representative images of immunofluorescence staining of MMP2 and MMP9 in cross-sections of the suprarenal aorta from each group (n=12). The scale bars in columns 1, 3, and 5 indicate 200 μm, and the ones in columns 2, 4, and 6 indicate 50 μm.

**Supplementary Figure 3**


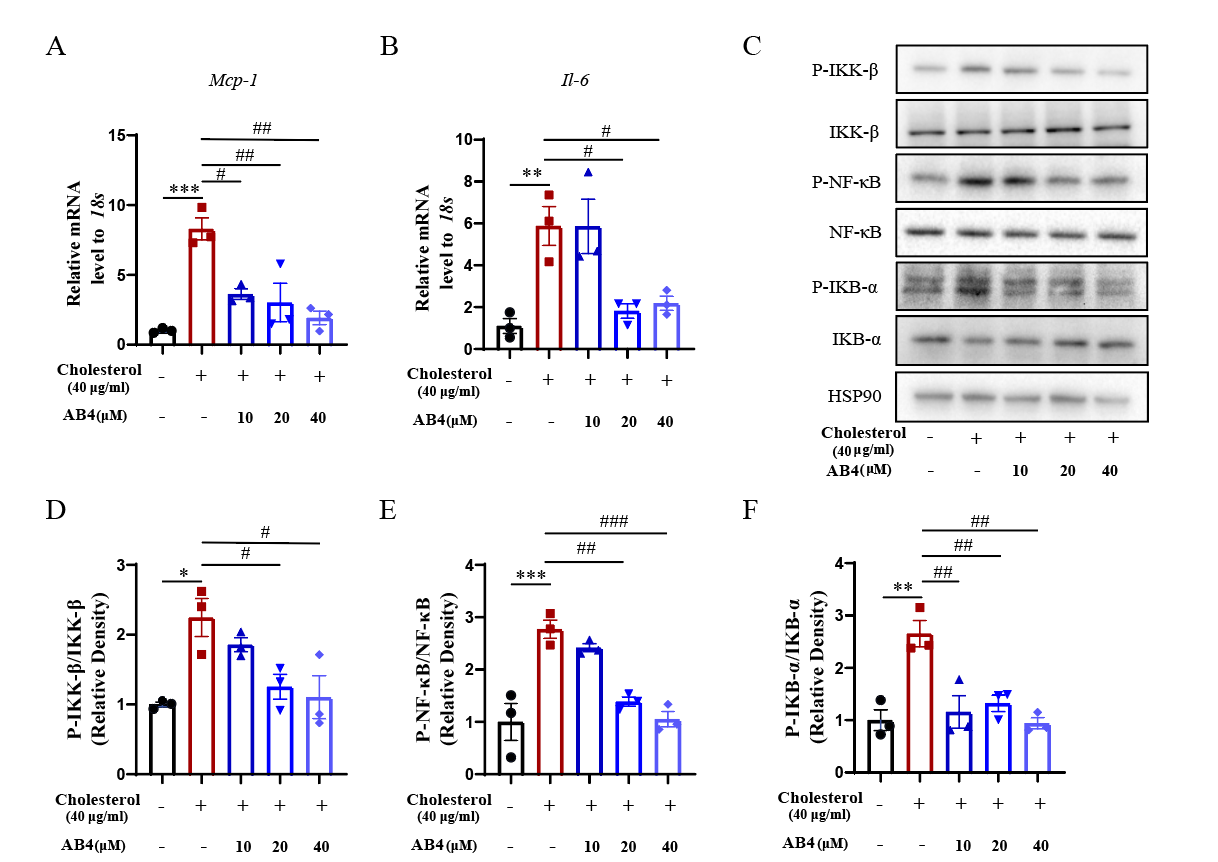


**Supplementary figure 3. AB4 decreases cholesterol-induced VSMC inflammation and NF-κB signaling pathway *in vitro*.**

**(A-B)** The relative mRNA levels of inflammatory cytokines *Mcp-1* and *Il-6* in VSMCs were examined by qRT-PCR (n=3); **(C)** NF-κB signaling pathway protein levels were examined by western blotting; **(D-F)**  Statistical differences were assessed by one-way ANOVA analysis of variance test. Results are presented as the mean ±SEM. *P < 0.05; **P < 0.01; ***P < 0.001 vs. Control group; ^#^P < 0.05; ^##^P < 0.01; ^###^P < 0.001 vs. Cholesterol group.

**Supplementary Figure 4**


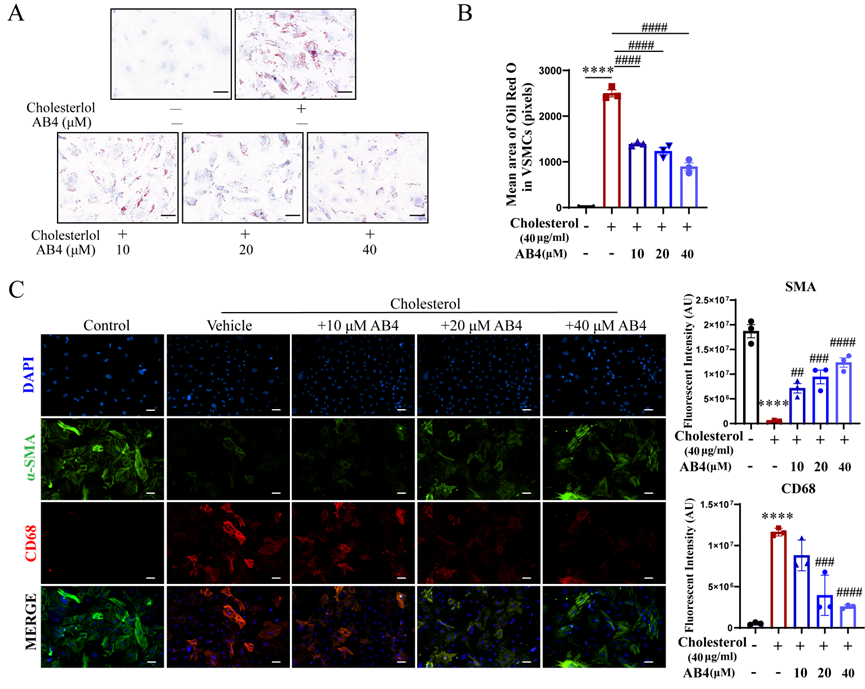


**Supplementary figure 4. AB4 inhibits VSMC transdifferentiation** ***in vitro*.**

**(A-B)** After 72 h cholesterol stimulation, VSMCs were washed three times with PBS buffer and fixed with 4% PFA for 30 min. Oil red O staining was performed for 30 mins. The representative images from each group are shown (**A**), and the quantification of the mean Oil red O area of each cell is shown (**B**), (n=3); **(C)** Fluorescence representative images of immunostaining of α-SMA (green), CD68 (red), and DAPI (blue) in different groups (left panel). The mean fluorescent intensity of SMA and CD68 were quantified (right panel), (n=3); The scale bar indicates 50 μm. Statistical defferences were assessed by one-way ANOVA analysis of variance test. Results are presented as the mean ± SEM. ****P < 0.0001vs. Control group; ^##^P < 0.01; ^###^P < 0.001; ^####^P < 0.0001 vs. Cholesterol group.

**Supplementary Figure 5**


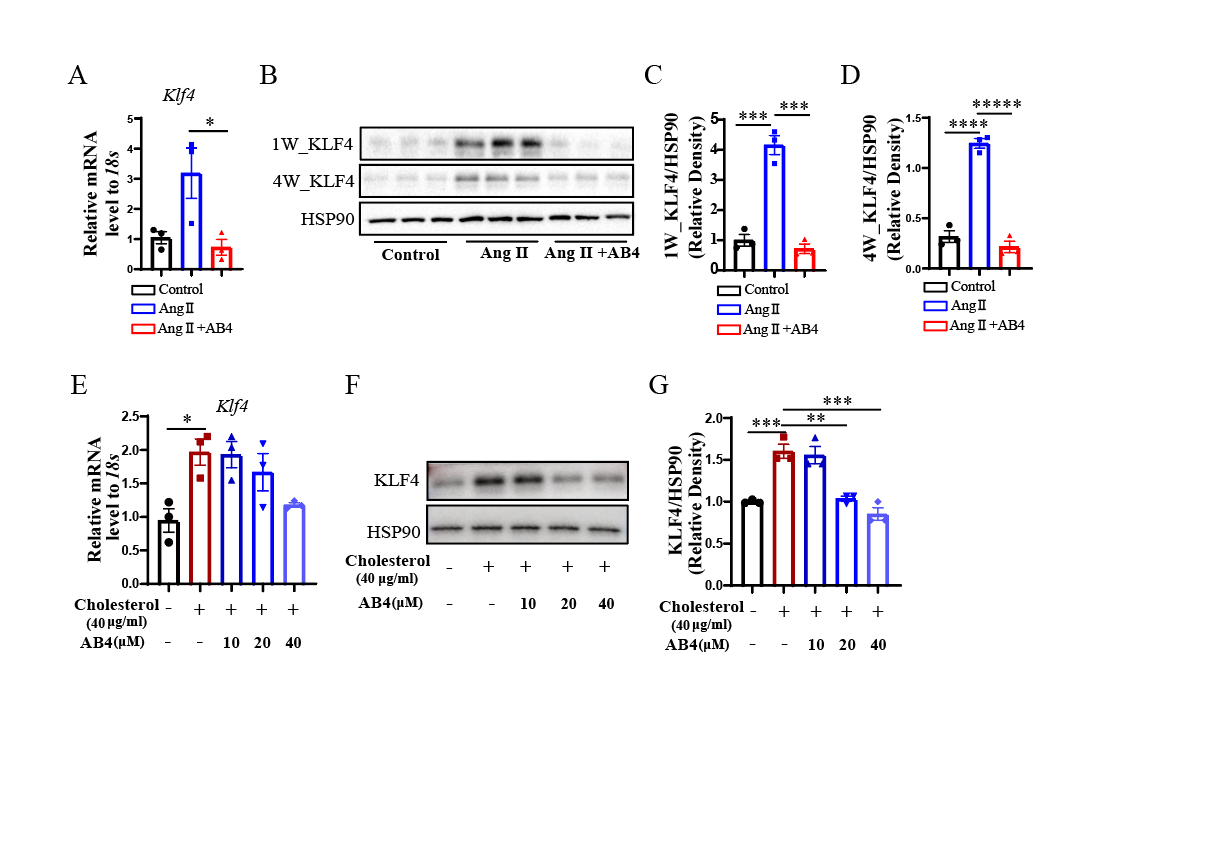


**Supplementary Figure 5. AB4 can treat abdominal aortic aneurysm formation and VSMC transdifferentiation in mice by regulating KLF4.**

**(A)** The relative mRNA level of *Klf4* in the aortae after Ang II stimulated for 4 weeks was measured by qRT-PCR (n=3); **(B)** Western blotting of KLF4 from aorta 1 and 4 weeks after Ang II stimulation; **(C-D)** KLF4 protein levels were quantified by densitometry analysis (n=3); **(E)** The relative mRNA level of *Klf4* in VSMCs treated with cholesterol with or without AB4 (n=3); **(F)** Western blotting of KLF4 from VSMCs treated with cholesterol and AB4; **(G)** KLF4 protein levels were quantified by densitometry analysis (n=3);. Statistical differences were assessed by one-way ANOVA analysis of variance test. Results are presented as the mean ± SEM. *P < 0.05; **P < 0.01; ***P < 0.001; ****P < 0.0001.

**Supplementary Figure 6**


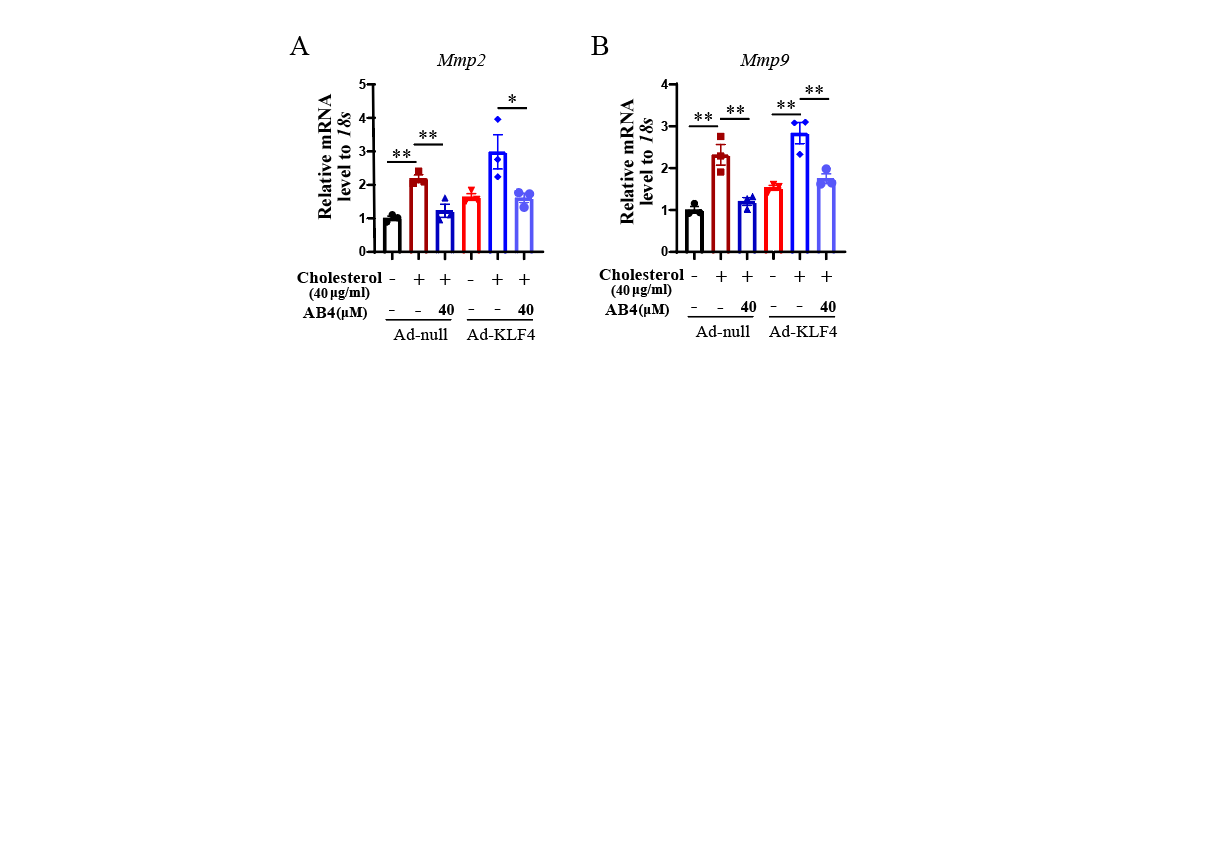


**Supplementary Figure 6.** **AB4 suppresses cholesterol induced MMPs transcription in a KLF4 independent manner in VSMC.**

**(A-B)** The relative mRNA levels of *Mmp2* and *Mmp9* after ad-null or ad-KLF4 infection in VSMCs were measured by qRT-PCR (n=3). Statistical differences were assessed by one-way ANOVA analysis of variance test. Data are presented as the mean ± SEM. *P < 0.05; **P < 0.01.

**Supplementary Table 1: Mouse Primer Sequences**

| Gene Name | Primer Sequence |
| --- | --- |
| *18S* | F: TTCCGATAACGAACGAGACTCT |
|  | R: TGGCTGAACGCCACTTGTC |
| *Mmp2* | F: CAAGTTCCCCGGCGATGTC |
|  | R: TTCTGGTCAAGGTCACCTGTC |
| *Mmp9* | F: CTGGACAGCCAGACACTAAAG |
|  | R: CTCGCGGCAAGTCTTCAGAG |
| *α-Sma* | F: CCCAACTGGGACCACATGG |
|  | R: TACATGCGGGGGACATTGAAG |
| *Sm22* | F: CAACAAGGGTCCATCCTACGG |
|  | R: ATCTGGGCGGCCTACATCA |
| *Myocd* | F: CAGGGAAGAAACCAGTGTCAG |
|  | R: GCTGCCAAACCATACTTGTCATT |
| *Tpm1* | F: CAGAAGGCCAAGTTCGACAG |
|  | R: GGAAGTCATATCGTTGAGAGCG |
| *Cnn1* | F: TCTGCACATTTTAACCGAGGTC |
|  | R: GCCAGCTTGTTCTTTACTTCAGC |
| *Myosin* | F: AAACGCCATGAGATGCCAC |
|  | R: CGGGCTTAGGCGATTCCTG |
| *Klf4* | F: GTGCCCCGACTAACCGTTG |
|  | R: GTCGTTGAACTCCTCGGTCT |
| *Cd68* | F: GGACCCACAACTGTCACTCAT |
|  | R: AAGCCCCACTTTAGCTTTACC |
| *Mac-2* | F: AGACAGCTTTTCGCTTAACGA |
|  | R: GGGTAGGCACTAGGAGGAGC |
| *Mcp-1* | F: TTAAAAACCTGGATCGGAACCAA |
|  | R: GCATTAGCTTCAGATTTACGGGT |
| *Cxcl10* | F: CCAAGTGCTGCCGTCATTTTC |
|  | R: GGCTCGCAGGGATGATTTCAA |
| *Tnf-α* | F: GACGTGGAACTGGCAGAAGAG |
|  | R: TTGGTGGTTTGTGAGTGTGAG |
| *Il-18* | F: GTGAACCCCAGACCAGACTG |
|  | R: CCTGGAACACGTTTCTGAAAGA |
| *Il-1β* | F: AGTTGACGGACCCCAAAAG |
|  | R: AGCTGGATGCTCTCATCAGG |
| *Il-6* | F: CCAAGAGGTGAGTGCTTCCC |
|  | R: CTGTTGTTCAGACTCTCTCCCT |

**Supplementary Table 2: Antibodies**

| Antibody | Vendor | Catalog number | Dilution |
| --- | --- | --- | --- |
| MMP9 | Abcam, UK | ab283575 | 1:500 |
| MMP2 | Abcam, UK | ab86607 | 1:500 |
| CNN1 | Abcam, UK | ab46794 | 1:1000 |
| α-SMA | Cell Signaling Technology, USA | 19245T | 1:1000 |
| SM22 | Abcam, UK | ab14106 | 1:1000 |
| KLF4 | Abcam, UK | ab214666 | 1:1000 |
| CD68 | Abcam, UK | ab125212 | 1:500 |
| MAC-2 | Abcam, UK | ab2785 | 1:1000 |
| p-IKK-β | Abcam, UK | ab194519 | 1:500 |
| IKK-β | Abcam, UK | ab32135 | 1:1000 |
| P-NF-κB | Cell Signaling Technology, USA | 3033s | 1:1000 |
| NF-κB | Cell Signaling Technology, USA | 6956S | 1:1000 |
| p-IKB-α | Abcam, UK | ab133462 | 1:1000 |
| IKB-α | CST, USA | 4814S | 1:1000 |
| MMP2  (immunofluorescence) | Proteintech, USA | 10373-2-AP | 1:200 |
| α-SMA  (immunofluorescence) | Sigma, USA | F3777 | 1:400 |
| CD68  (immunofluorescence) | BioRad, USA | MCA1957 | 1:100 |
